# The International Conference on Intelligent Biology and Medicine (ICIBM) 2018: genomics with bigger data and wider applications

**DOI:** 10.1186/s12864-018-5369-3

**Published:** 2019-02-04

**Authors:** Zhijin Wu, Jingwen Yan, Kai Wang, Xiaoming Liu, Yan Guo, Degui Zhi, Jianhua Ruan, Zhongming Zhao

**Affiliations:** 1 Department of Biostatistics, Brown University, Providence, RI 02912 USA; 2 Department of Biohealth Informatics, Indiana University Purdue University Indianapolis, Indianapolis, IN 46202 USA; 3 Raymond G. Perelman Center for Cellular and Molecular Therapeutics, Children’s Hospital of Philadelphia, Philadelphia, PA 19104 USA; 4 Department of Pathology and Laboratory Medicine, University of Pennsylvania, Philadelphia, PA 19104 USA; 5 College of Public Health, University of South Florida, Tampa, FL 33612 USA; 6 Comprehensive Cancer Center, University of New Mexico, Albuquerque, NM 87131 USA; 7 Center for Precision Health, School of Biomedical Informatics, The University of Texas Health Science Center at Houston, Houston, TX 77030 USA; 8 Department of Computer Science, The University of Texas at San Antonio, San Antonio, TX 78249 USA

## Abstract

The sixth International Conference on Intelligent Biology and Medicine (ICIBM) took place in Los Angeles, California, USA on June 10–12, 2018. This conference featured eleven regular scientific sessions, four tutorials, one poster session, four keynote talks, and four eminent scholar talks. The scientific program covered a wide range of topics from bench to bedside, including 3D Genome Organization, reconstruction of large scale evolution of genomes and gene functions, artificial intelligence in biological and biomedical fields, and precision medicine. Both method development and application in genomic research continued to be a main component in the conference, including studies on genetic variants, regulation of transcription, genetic-epigenetic interaction at both single cell and tissue level and artificial intelligence. Here, we write a summary of the conference and also briefly introduce the four high quality papers selected to be published in BMC Genomics that cover novel methodology development or innovative data analysis.

## Introduction

The 2018 International Conference on Intelligent Biology and Medicine (ICIBM 2018) was held from June 10th to 12th, 2018 in Los Angeles, California, USA. This is the sixth ICIBM conference and it became the first official conference of The International Association for Intelligent Biology and Medicine (IAIBM). Since its inception in 2012, ICIBM conference series aim to: 1) foster interdisciplinary and multidisciplinary research in bioinformatics, systems biology, intelligent computing/artificial intelligence, bioengineering, and data sciences, and 2) offer an educational program for trainees and young investigators in multiple scientific disciplines to learn or exchange the new methods/tools/discoveries in these areas and to build a professional network among both the established and junior investigators.

We have expanded our ICIBM conference each year since 2012. The summary of the previous conferences is reported in previous introduction article [1–5]. The ICIBM 2018 was carefully prepared and attracted a record number of the paper submission and participants, and was the largest ICIBM conference. Specifically, we received 95 original manuscripts and 44 abstracts in the areas of bioinformatics, genomics, systems biology, machine learning, and biomedical informatics. Each paper was carefully reviewed by the program committee members. Among these submissions, we observed more papers coming out from emerging fields such as deep learning, new genome technologies, and big medical data science. Accordingly, our keynote speeches and scientific sessions reflect these trends. In addition, financial support from National Science Foundation allowed us to support 20 trainees in diverse background and across the world to attend and present their works in the ICIBM conference. As before, the Award Committee carefully reviewed travel award applications based on the quality of the research (paper or abstract), the financial need, and diversity/minority of the attendees. Below, we briefly summarize the scientific program of the ICIBM 2018 and also provide an introduction of the 4 research articles selected for this BMC Genomics supplement issue.

## Overall scientific program of ICIBM 2018

The ICIBM 2018 scientific program includes eleven regular scientific sessions, four tutorials, one poster session, four keynote talks, and four eminent scholar talks. It covered a variety of the topics in bioinformatics, systems biology, machine learning, data sciences, and biomedical informatics.

### Keynote and eminent scholar lectures

Four distinguished scientists were invited to deliver the keynote speeches to the ICIBM attendees. These speakers were: (1) Joshua C. Denny, MD, MS, a Professor of Biomedical Informatics and Medicine, Director of the Center for Precision Medicine and Vice President of Personalized Medicine at Vanderbilt University Medical Center. He is a Fellow of the American College of Medical Informatics and a member of the National Academy of Medicine. He presented “Huge cohorts, genomics, and clinical data to personalize medicine”. In his talk, Dr. Denny demonstrated how electronic health records (EHRs) and genomic data can be combined for phenome-wide association studies, leading to personalized medicine. He also introduced the era of huge international cohorts such as the UK Biobank, Million Veteran Program, and the newly started *All of Us* Research Program, which will make millions of individuals available with dense molecular and phenotypic data. (2) Paul D. Thomas, Ph.D., an Associate Professor in the Preventive Medicine Department, and who heads the Bioinformatics Division at the University of Southern California Keck School of Medicine. Dr. Thomas is the leader of the Gene Ontology project, which is among the world’s largest bioinformatics projects. In Dr. Thomas presentation entitled “Reconstructing the large-scale evolution of genomes and gene functions”, he gave an overview of the reconstruction methods and the related findings, introduced his work that reconstructed the evolutionary history of over 1 million genes in 15,000 gene families covering all domains of life, and also his work inferring human gene function from experiments in “model organisms” such as the fruit fly and yeast.

In the third keynote speech, Dr. Alexander Hoffmann, a Professor of Microbiology, Immunology, and Molecular Genetics, and Director of the Institute for Quantitative and Computational Biosciences (QCB) at UCLA, demonstrated “Learning how to predict immune responses”. He discussed how molecular network dynamics and molecular noise affect immune cell function, and some of the modeling strategies that allow for prediction and insight. The fourth keynote speaker was Dr. Jason Moore, who holds the Edward Rose Professor of Informatics and Director of the Penn Institute for Biomedical Informatics. He also serves as Senior Associate Dean for Informatics and Chief of the Division of Informatics in the Department of Biostatistics, Epidemiology, and Informatics. Dr. Moore is a fellow of the American Association for the Advancement of Science (AAAS), the American College of Medical Informatics (ACMI), the American Statistical Association (ASA), and a Kavli fellow of the National Academy of Sciences. In his presentation, entitled “Accessible artificial intelligence for data science”, Dr. Moore introduced the history of artificial intelligence (AI) and then demonstrated the PennAI, an accessible, open-source, and user-friendly AI system at the University of Pennsylvania. PennAI brings AI and automated machine learning technology to everyone who wants to incorporate this technology into their big data analytics agenda.

ICIBM 2018 also featured four eminent scholar talks. These talks were delivered by four renowned researchers in their specific fields. (1) Dr. Xinghua Lu, a professor in the Department of Biomedical Informatics, University of Pittsburgh, presented “From big data to bedside (BD2B): Precision oncology in an era of artificial intelligence”. (2) Ting Wang, Ph.D., an Associate Professor of Genetics, Computer Science and Engineering, and Biostatistics, and the Director of Computational and Systems Biology Program at Washington University School of Medicine, talked about “Exploring the dark matter in genomics data”. Dr. Xinshu (Grace) Xiao, a Professor and Vice Chair of the Department of Integrative Biology and Physiology at the University of California, Los Angeles, gave the presentation entitled “Deciphering the function of single-nucleotide variants in the RNA”. Finally, Dr. Charles Wang, the founding Director of the Center for Genomics and a professor in the School of Medicine, Loma Linda University (LLU), gave the lecture focusing on “Vegetarian diet-modulated epigenetic reprogramming and longevity”.

### ICIBM tutorials

As before, ICIBM offered tutorials to help trainees or other investigators learn the frontier informatics technologies. ICIBM 2018 provided four tutorials: Deep architectures are not necessary for anomaly classification of matrix-formed omics data (offered by Dr. Yan Guo, Associate & Endowed Professor in Cancer Bioinformatics and Computational Biology, Director of Bioinformatics Shared Resources, Comprehensive Cancer Center University of New Mexico), Single-cell sequencing analysis (offered by Dr. Chi Zhang, Indiana University School of Medicine, and Dr. Xiao Dong, Albert Einstein College of Medicine), Workshop on 3D genome organization (offered by Dr. Feng Yue, Director, Bioinformatics Division, Institute for Personalized Medicine, Penn State College of Medicine), and WashU Epigenome Browser (offered by Dr. Ting Wang, Associate Professor of Genetics and Computer Science and Engineering, Washington University at St. Louis). All the tutorial lecturers are the experts in the related techniques or tools, or the developers of the tools.

### Scientific sessions

ICIBM 2018 included eleven concurrent scientific sessions and one poster session. Speakers in the regular sessions were chosen from those top ranked manuscripts after peer review. The topics in these session covered bioinformatics, genomics, systems biology, intelligent computing, data sciences, computational drug discovery, and biomedical informatics. To promote international collaboration, we first time organized an International PI talk session. Several principal investigators (PIs) from oversea presented their exciting projects in this session followed by extensive discussion. In addition, two best papers were selected and honored in the conference. The eleven session are:

- ◦ Session I: NGS & Tools I
- ◦ Session II: Systems Biology I
- ◦ Session III: Bioinformatics
- ◦ Session IV: NGS & Tools II
- ◦ Session V: Systems Biology II
- ◦ Session VI: Medical Informatics
- ◦ Session VII: Cancer Genomics I
- ◦ Session VIII: Systems Biology III
- ◦ Session IX: Computational Drug Discovery
- ◦ Session X: International PI Talk
- ◦ Session XI: Cancer Genomics II

## The science program for the ICIBM 2018 genomics track

Since 2010, ICIBM meetings have been covering the latest developments in the entire pipeline of genomics research from low level data processing, to modeling, prediction and visualization, as well as to application to the real data sets. The tasks have grown from single feature to network analysis, and the scale of studies have grown from single data set to joint analysis of large scale cohorts or integration of data from multiple types and sources. The ICIBM 2018 reflects the trend to harvesting signal from increasing amount of data and keeping the development of computational methods to meet the needs of emerging biotechnology that presents new data types. Below, we summarize the contribution of ten papers included in this supplement issue.

The paper by Du et al. [6] explored the idea of gene embedding, distributed representation of genes, in the spirit of word embedding. The authors described a machine learning method that uses transcriptome-wide gene co-expression patterns from 984 publicly available data sets to generate a distributed representation of genes. They trained a vector representation (vectors of dimension 200) of all human genes, which captured functional relatedness of genes in terms of recovering known-pathways. The usefulness of the embedded gene vectors is illustrated in tasks such as gene-gene interaction prediction based solely on gene names.

RNA-sequencing (RNA-seq) has now become a routine technique in genomic studies and its application has expanded beyond quantifying transcription activity. Mohammad et al. [7] introduced a cell line identification method using variants derived from RNA-seq data. They calculated pair-wise correlations based on frequencies and depth of coverage values of variants and conducted comparative analysis of correlations across cell lines. The results showed substantial difference in variant profiles between different cell lines. On the other hand, identical, synonymous and derivative cell lines could share high variant identity and were highly correlated. Their identification method, CeL-ID, has high accuracy of identifying the cell line when a pure cell line is involved, and can be used to detect cross-contamination with a mixture model.

Emerging technologies that bring in new data types have become a constant phenomenon in the genomics era. The paper by Liu et al. [8] presents a computational method to detect DNA modification from single-molecule sequencing data generated by Nanopore technology. This method, named NanoMod, takes Nanopore sequencing data on paired DNA samples with and without modifications, and identifies modified bases by contrasting the signal distributions in the two samples and adjusting for local neighborhood effects. Not only does NanoMod show improved performance in the detection of DNA modification in simulation studies, but also its ability to detect de novo modification without training data makes it particularly desirable.

In addition to technology advancement, innovative analysis using existing datasets continue to bring discoveries. Pei et al. [9] investigated multi-trait associations using genome-wide association studies (GWAS) datasets. Specifically, the authors applied pathway-based analysis of GWAS summary statistics with an analytical framework for systematic integration of cross-trait associations. They used 25 traits belonging to four phenotype groups. The pathway-based analysis provided increased power to estimate cross-trait associations compared to gene-level analysis. The study revealed that the risk variants to the 25 different traits aggregated in particular biological pathways and that these pathways were frequently shared among traits. The results confirmed known mechanisms and also suggested several novel insights into the etiology of multi-traits.

## Conference organization

### 2018 International conference on intelligent biology and medicine (ICIBM 2018)

(June 10–12, 2018 Los Angeles, California, USA)

We would like to express our sincere gratitude to the members of the Steering, Program, Publication, Workshop/Tutorial, Publicity, Award, Trainee and Local Organization Committees, as well as to all the reviewers, volunteers and invited speakers, who spent their valuable time and effort on making ICIBM 2018 a success. The conference accomplishments are the results of support and hard work of all these people.

#### **Hosts and sponsors**

The International Association for Intelligent Biology and Medicine (IAIBM), The University of Texas Health Science Center at Houston (UTHealth), National Science Foundation, UTHealth Center for Precision Health, and UTHealth Data Science and Informatics Core for Cancer Research.

**General Chair** Zhongming Zhao (The University of Texas Health Science Center at Houston).

**Steering Committee** Yidong Chen (The University of Texas Health Science Center at San Antonio), Kun Huang (Indiana University), Tony Hu (Drexel University), Lang Li (The Ohio State University), Yi Xing (University of California Los Angeles), Jiajie Zhang (The University of Texas Health Science Center at Houston), Wenjin J. Zheng (The University of Texas Health Science Center at Houston).

**Program Committee** Chair: Kai Wang (Children’s Hospital of Philadelphia), Co-Chair: Degui Zhi (The University of Texas Health Science Center at Houston). Members: Genevera Allen (Rice University), Jianlin Cheng (University of Missouri Columbia), Luca Giancardo (The University of Texas Health Science Center at Houston), Assaf Gottlieb (The University of Texas School of Biomedical Informatics at Houston), Mike Guo (University of New Mexico), Yan Guo (University of New Mexico), Zhi Han (Indiana University School of Medicine), Matthew Hayes (Xavier University of Louisiana), Kun Huang (The Ohio State University), Weichun Huang (National Institute of Environmental Health Sciences), Yang Huang (Kaiser Permanente), Yufei Huang (University of Texas at San Antonio), Zhi-Linag Ji (Xiamen University, China), Peilin Jia (The University of Texas Health Science Center at Houston), Victor Jin (University of Texas at San Antonio), Yufang Jin (University of Texas at San Antonio), Jun Kong (Emory University), Dmitry Korkin (Worcester Polytechnic Institute), Rui Kuang (University of Minnesota Twin Cities), K.B. Kulasekera (University of Louisville), Qiwei Li (Rice University), Fuhai Li (The Ohio State University), Tao Li (Nankai University, China), Zhaohui Li (Nankai University, China), Aimin Li (Xi’an University of Technology, China), Ping Liang (Brock University, Canada), Liao Li (University of Delaware), Honghuang Lin (Boston University), Nan Liu (Chinese Academy of Sciences, China), Yin Liu (The University of Texas Health Science Center at Houston), Xiaoming Liu (The University of Texas Health Sciences Center at Houston), Yunlong Liu (Indiana University), Zhandong Liu (Baylor College of Medicine), Zhiyong Lu (NCBI, National Library of Medicine, NIH), Mirjana Maletic (Baylor College of Medicine), Patricio A Manque (Universidad Mayor, Chile), Huaiyu Mi (University of Southern California), Nitish Mishra (University of Nebraska Medical Center), Qiangxing Mo (Baylor College of Medicine), Tabrez Mohammad (The University of Texas Health Science Center at San Antonio), Hatice Ozer (Ohio State University), Ranadip Pal (Texas Tech University), Jiang Qian (Johns Hopkins University), Guimin Qin (Xidian University), Thomas Rindflesch (National Institutes of Health), Jianhua Ruan (The University of Texas at San Antonio), Bairong Shen (Soochow University, China), Xiaofeng Song (Nanjing University of Aeronautics and Astronautics, China), Fengzhu Sun (University of Southern California), Wing-Kin Sung (National University of Singapore, Singapore), Manabu Torii (Kaiser Permanente), Ying-Wooi Wan (Baylor College of Medicine), Jun Wan (Indiana University), Yufeng Wang (University of Texas at San Antonio), Junbai Wang (Radium Hospital), Qingguo Wang (Memorial Sloan Kettering Cancer Center), Jiaying Wang (Xian Jiaotong University, China), Chaochun Wei (Shanghai Jiao Tong University, China), Xiwei Wu (City of Hope National Medical Center), Yonghui Wu (University of Florida), Zhijin Wu (Brown University), Junfeng Xia (Anhui University, China), Lu Xie (Shanghai Center for Bioinformation Technology, China), Lei Xie (City University of New York), Hua Xu (The University of Texas Health Science Center at Houston), Jianhua Xuan (Virginia Tech), Yu Xue (Huazhong University of Science and Technology, China), Zhenqing Ye (The University of Texas Health Science Center at San Antonio), Sungroh Yoon (Seoul National University, Korea), Feng Yue (Penn State University), Habil Zare (Texas State University), Rui Zhang (University of Minnesota), Shaojie Zhang (University of Central Florida), Han Zhang (Nankai University, China), Bing Zhang (Baylor College of Medicine), Zhongming Zhao (The University of Texas Health Science Center at Houston), Min Zhao (University of the Sunshine Coast, Australia), Jim W. Zheng (The University of Texas Health Science Center at Houston), Xianghong Zhou (University of California Los Angeles), Yunyun Zhou (University of Mississippi).

**Publication Committee** Chair: Zhijin Wu (Brown University), Co-Chair: Jianhua Ruan (The University of Texas at San Antonio).

**Workshop/Tutorial Committee** Chair: Feng Yue (Pennsylvania State University), Co-Chair: Yan Guo (University of New Mexico).

**Publicity Committee** Chair: Lana Garmire (University of Hawaii), Co-Chair: Sun Kim (Seoul National University, Korea), Co-Chair: Yu Xue (Huazhong University of Science and Technology, China).

**Award Committee** Chair: Fuhai Li (Ohio State University), Co-Chair: Lei Xie (City University of New York).

**Trainee Committee** Chair: Abolfazl Doostparast (Columbia University), Co-Chair: Qian Liu (Children’s Hospital of Philadelphia).

**Local Organization Committee** Chair: Jessica Li (University of California Los Angeles), Co-Chair: Matteo Pellegrini (University of California Los Angeles), Co-Chair: Xiaoming Liu (The University of Texas Health Science Center at Houston).
